# Judging Statistical Models of Individual Decision Making under Risk Using In- and Out-of-Sample Criteria

**DOI:** 10.1371/journal.pone.0102269

**Published:** 2014-07-16

**Authors:** Andreas C. Drichoutis, Jayson L. Lusk

**Affiliations:** 1 Agricultural Economics and Rural Development, Agricultural University of Athens, Athens, Greece; 2 Agricultural Economics, Oklahoma State University, Stillwater, Oklahoma, United States of America; Middlesex University London, United Kingdom

## Abstract

Despite the fact that conceptual models of individual decision making under risk are deterministic, attempts to econometrically estimate risk preferences require some assumption about the stochastic nature of choice. Unfortunately, the consequences of making different assumptions are, at present, unclear. In this paper, we compare three popular error specifications (Fechner, contextual utility, and Luce error) for three different preference functionals (expected utility, rank-dependent utility, and a mixture of those two) using in- and out-of-sample selection criteria. We find drastically different inferences about structural risk preferences across the competing functionals and error specifications. Expected utility theory is least affected by the selection of the error specification. A mixture model combining the two conceptual models assuming contextual utility provides the best fit of the data both in- and out-of-sample.

## Introduction

Virtually all conceptual models of risky choice, including Expected Utility Theory (EUT) and the behavioral alternatives such as prospect theory, are deterministic. The deterministic nature of the theories presents a challenge for applied economists attempting to econometrically estimate risk preferences in a sample of individuals. In essence, the analyst must make assumptions about the decision making process that go above and beyond the content of the theory, making it difficult to conduct clean tests of the underlying theory itself and to confidently identify underlying structural parameters. The literature on stochastic error specifications is not negligible but is by no means a large one. While a few previous studies have analyzed the extent to which different stochastic error specifications influence estimates of risk preferences [1], [2], there have been new developments in the field [3] that have not been thoroughly addressed in previous model comparisons, and there has been an almost exclusive focus on the ability of models to fit the data in-sample (with few exceptions) over the recent years.

The focus on in-sample fit is particularly important in determining which decision making theory, EUT or a behavioral alternative, best describes lottery choices. EUT is a relatively parsimonious theory, characterizing risk preferences simply by the curvature of the utility function over income or wealth. Some popular functional forms such as constant relative (or constant absolute) risk aversion consist of a single parameter. Behavioral theories often proceed by adding parameters to the basic EUT set-up. Cumulative prospect theory, for example, allows for different degrees of curvature in the gain and loss-domains and for additional parameters describing the extent to which individuals under- or over-weight low probability events (both in the gain and loss domains). Given the additional parameters, there might be a tendency for such behavioral models to over-fit the data, and while in-sample test statistics, such as Akaike or Bayesian Information Criteria, suggest improvements in model fit, this is no guarantee the model will perform better predicting out-of-sample.

Starting with [4] and [5], several previous studies have compared different decision making models under risk. Table 1 lists these empirical studies in chronological order. The literature has attempted to evaluate several different decision theories albeit EUT and Rank Dependent Utility (RDU) are the main contenders. Various error stories have been evaluated as well, using a variety of model fit criteria. The early literature frequently relied on Akaike's Information Criterion (AIC) [6], while it is only recently that there has been a focus on the predictive accuracy of the models using out-of-sample fit criteria. The table does not list all the utility functions (e.g., constant relative risk aversion, expo-power etc.) and probability weighting functions that each study has evaluated under different error stories because this would render the table unreadable.

**Table 1:** Literature on error stories.

Study	Decision theory	Error story	Model fit criteria
[36]	EUT	Trembles, Fechner error (Logit link), Random preferences	Akaike Information Criterion
[37]	Expected value, EUT, RDU, Disappointment aversion theory, Prospective reference theory, Quadratic utility, Regret with independence, Weighted utility	Trembles, Fechner	Akaike Information Criterion
[38]	EUT, RDU, Prospective reference theory, Weighted utility, Quadratic utility, Regret with independence	Homoscedastic errors, Variants of heteroscedastic errors	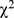 tests, Akaike Information Criterion
[39]	EUT, RDU	Fechner error (Probit link), Fechner error (Probit link) with trembles, Random preferences with trembles	Log-likelihood, Likelihood ratio tests, Vuong's test
[40]	Cumulative PT	Trembles, Fechner error (Probit link), Fechner error (Logit link), Luce error	Akaike Information Criterion
[41]	Cumulative PT, Stochastic EUT	Fechner error (Probit link), Fechner error (Logit link), Truncated random errors	Log-likelihood, Vuong's test
[12], [13]	EUT, RDU	Fechner error (Logit link), Luce error, Contextual utility (Logit link), Wandering vector model, Random preferences	Log-likelihood, Vuong's test, Out-of-sample fit
[42]	Expected value, EUT, Choquet EUT, PT, Cumulative PT, Decision Field Theory, [43] MaxMin EUT, [43] MaxMax EUT, [44], Alpha theory, MaxMin, MaxMax, Minimum Regret	Trembles, Luce error, Fechner error, Contextual utility, Wandering vector model, Random preferences, Variance of the valence difference	Out-of-sample fit
[45]	Expected value, EUT, Regret theory, Skew-symmetric bilinear utility theory, Yaari's Dual theory, Disappointment aversion theory	Trembles, Fechner error (Probit link), Fechner with heteroskedastic errors, Fechner with truncated errors, Random utility, Luce error	Likelihood ratio tests, Vuong's test
[3]	EUT, RDU	Fechner error (logit link), Luce error, Contextual utility (logit link), Random preferences	Log-likelihood, Vuong's test, Out-of-sample fit

Notes: EUT = Expected utility theory, RDU = Rank Dependent utility, PT = Prospect theory. In some papers ‘trembles’ are also called ‘constant error probability model’ and the ‘Fechner error’ is also called ‘white noise’. The [42] paper is about uncertainty, not risk. In the studies listed above, different decision theories are combined with different error stories; not all combinations are possible though. For example, ‘variance of the valence difference’ is specific to Decision Field Theory. The specific papers should be advised for more details.

Because most experimental studies are performed with a relatively small sample of subjects, it would seem that most analysts are attempting to extrapolate risk preferences out-of-sample to the more general population, and as such, studying out-of-sample prediction performance appears a worthwhile line of inquiry. Judging out-of-sample prediction performance is not always easy for discrete choice problems, and as such, we turn to the out-of-sample-log-likelihood function approach long used in the marketing literature for model selection [7], [8] which has been further elucidated in the economics literature [9], [10].

The purpose of this paper is to use several in- and out-of sample model selection criteria to determine which stochastic error specification and decision theory best fits lottery choice data gathered in an experimental setting. In particular, we compare three different error specifications: one of those has been used by [5] and is called the Fechner error specification (or sometimes called ‘white noise’); the second error specification, called the Luce error, has been popularized by [11] (H&L); the third error specification has been recently introduced by [3], [12], [13] and given the name contextual utility. Obviously, this is not an exhausitive list of all possible errors specifications. On the contrary, we picked (what seems to us) the most popular error stories in the relevant literature. Similarly, we focus on just two decision theories: EUT and RDU. Although there has been a burst of theoretical modeling, which has resulted in a long list of decision theories, EUT and RDU theory remain the leading alternatives for the description of behavior under risk. Therefore, we focus our attention to these theories alone. The methods we describe herein can be extended to a larger battery of decision theories and error specifications.

We extend previous studies in two ways. First, instead of testing whether subjects' choices adhere to one decision theory alone (i.e., EUT or RDU) we allow more than one data generating processes (i.e., combining EUT and RDU). These combined models have been called mixture specifications in the respective literature [14], [15]. We further test how these mixture models combine with different error specifications. Second, we add to the list of battery tests a non-parametric alternative to the Vuong's non-nested test. Overall, our battery of tests can provide a better characterization of which decision theory and error story is more likely to be the ‘correct’ one.

The next section of the paper describes the laboratory experiment we conducted to elicit preferences for competing lotteries. Then, we describe the competing approaches used to estimate risk preferences, after which we present the results from the competing models. Following this discussion, we discuss different model selection criteria and indicate the best fitting models. The last section concludes.

## Methods

### Ethics statement

The data in this study have been collected from the undergraduate population of the University of Ioannina while one of the authors (Andreas C. Drichoutis) was still a faculty member of the Economics department. The University does not operate an ethical review board and as such no consideration, approval or waiver was possible to obtain. There is a university ethics code that lays out general principles that experimenters should abide by but no review board is responsible for checking whether projects abide by the code. This is the case with all non-invasive studies that are being conducted in the premises of the university or on behalf of the university. Subjects did give an oral consent for participating in the study before each experimental session. When checking subjects' name against a list of pre-registered participants, the experimenter asked each person individually whether s/he wants to participate in the experiment, informed the subject that the experiment only involves data collection and emphasized that s/he is free to leave if s/he wants to. In addition, the data collection was completely anonymized (as we describe below) and no data can be linked to any single person.

### Description of the experiment

A conventional lab experiment was conducted using the z-Tree software [16]. Subjects consisted of undergraduate students at the University of Ioannina, Greece and were recruited using the ORSEE recruiting system [17]. During the recruitment, subjects were told that they would be given the chance to make more money during the experiment. More specifically, subjects were told that “In addition to a fixed fee of € 10, you will have a chance of receiving additional money up to € 25. This will depend on the decisions you make during the experiment.” [18] have shown that stochastic and non-stochastic fees can significantly affect self-selection of subjects with respect to risk attitudes.

Subjects participated in sessions of group sizes that varied from 9 to 11 subjects per session (all but two sessions involved groups of 10 subjects). In total, 100 subjects participated in 10 sessions that were conducted between December 2011 and January 2012. Each session lasted about 45 minutes and subjects were paid a € 10 participation fee. Subjects were given a power point presentation explaining the lottery choice tasks as well as printed copies of instructions. They were also initially given a five-choice training task to familiarize them with the choice screens that would appear in the tasks involving real payouts. Subjects were told that choices in the training phase would not count toward their earnings and that this phase was purely hypothetical.

Full anonymity was ensured by asking subjects to choose a unique three-digit code from a jar. The code was then entered at an input stage once the computerized experiment started. The experimenter only knew correspondence between digit codes and profits. Profits and participation fees were put in sealed envelopes (the digit code was written on the outside) and were exchanged with digit codes at the end of the experiment. No names were asked at any point of the experiment. Subjects were told that their decisions were independent from other subjects, and that they could finish the experiment at their own convenience. Average total payouts including lottery earnings were € 15.2 (S.D. = 4.56).

### Risk preference elicitation

We elicited risk preferences using the popular H&L [11] Multiple Price List (MPL) task, at two payout (low versus high) amounts. Note that, the data analyzed in this paper are a subset of the data collected in the experiment. Data analysis of the full dataset with a different research focus is reported in [19]. The experiment contained different formats of risk preference elicitation tasks. Here we analyze the data coming from the more popular H&L task. The baseline H&L MPL presented subjects with a choice between two lotteries, A or B, as illustrated in Table 2. In the first row, the subject was asked to make a choice between lottery A, which offers a 10% chance of receiving € 2 and a 90% chance of receiving € 1.6, and lottery B, which offers a 10% chance of receiving € 3.85 and a 90% chance of receiving € 0.1. The expected value of lottery A is € 1.64 while for lottery B it is € 0.475, which results in a difference of € 1.17 between the expected values of the lotteries. Proceeding down the table to the last row, the expected values of both lotteries increase, but the rate of increase is larger for option B. For each row, a subject choose A or B, and one row was randomly selected as binding for the payout. The last row is a simple test of whether subjects understood the instructions correctly. In fact, 16 out of 100 subjects failed to pass this test concerning comprehension of lotteries and were omitted from our sample. The high payout task is identical to the control (shown in Table 2) except that all payouts are scaled up by a magnitude of five.

**Table 2:** The H&L Multiple Price List.

Lottery A				Lottery B				EV 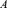 (€)	EV 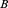 (€)	Difference (€)	Open RRA coefficient interval if subject switches to Lottery B (assumes EUT)	
*p*	€	*p*	€	*p*	€	*p*	€					
0.1	2.00	0.9	1.60	0.1	3.85	0.9	0.10	1.640	0.475	1.17	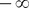	−1.71
0.2	2.00	0.8	1.60	0.2	3.85	0.8	0.10	1.680	0.850	0.83	−1.71	−0.95
0.3	2.00	0.7	1.60	0.3	3.85	0.7	0.10	1.720	1.225	0.50	−0.95	−0.49
0.4	2.00	0.6	1.60	0.4	3.85	0.6	0.10	1.760	1.600	0.16	−0.49	−0.15
0.5	2.00	0.5	1.60	0.5	3.85	0.5	0.10	1.800	1.975	−0.18	−0.15	0.14
0.6	2.00	0.4	1.60	0.6	3.85	0.4	0.10	1.840	2.350	−0.51	0.14	0.41
0.7	2.00	0.3	1.60	0.7	3.85	0.3	0.10	1.880	2.725	−0.85	0.41	0.68
0.8	2.00	0.2	1.60	0.8	3.85	0.2	0.10	1.920	3.100	−1.18	0.68	0.97
0.9	2.00	0.1	1.60	0.9	3.85	0.1	0.10	1.960	3.475	−1.52	0.97	1.37
1	2.00	0	1.60	1	3.85	0	0.10	2.000	3.850	−1.85	1.37	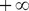

Notes: EV 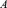 stands for expected value of lottery A, EV 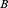 stands for expected value of lottery B, CRRA stands for constant relative risk aversion, EUT stands for expected utility theory. The last two columns show the implied coefficient of RRA interval. For example, if a person chooses for the first three rows lottery option A and then chooses lottery option B, his/her implied coefficient of RRA (assuming EUT) would be in the interval of [−0.49,−0.15]. Last four columns showing expected values and implied coefficient of RRA intervals were not shown to subjects.

**Figure 1:**
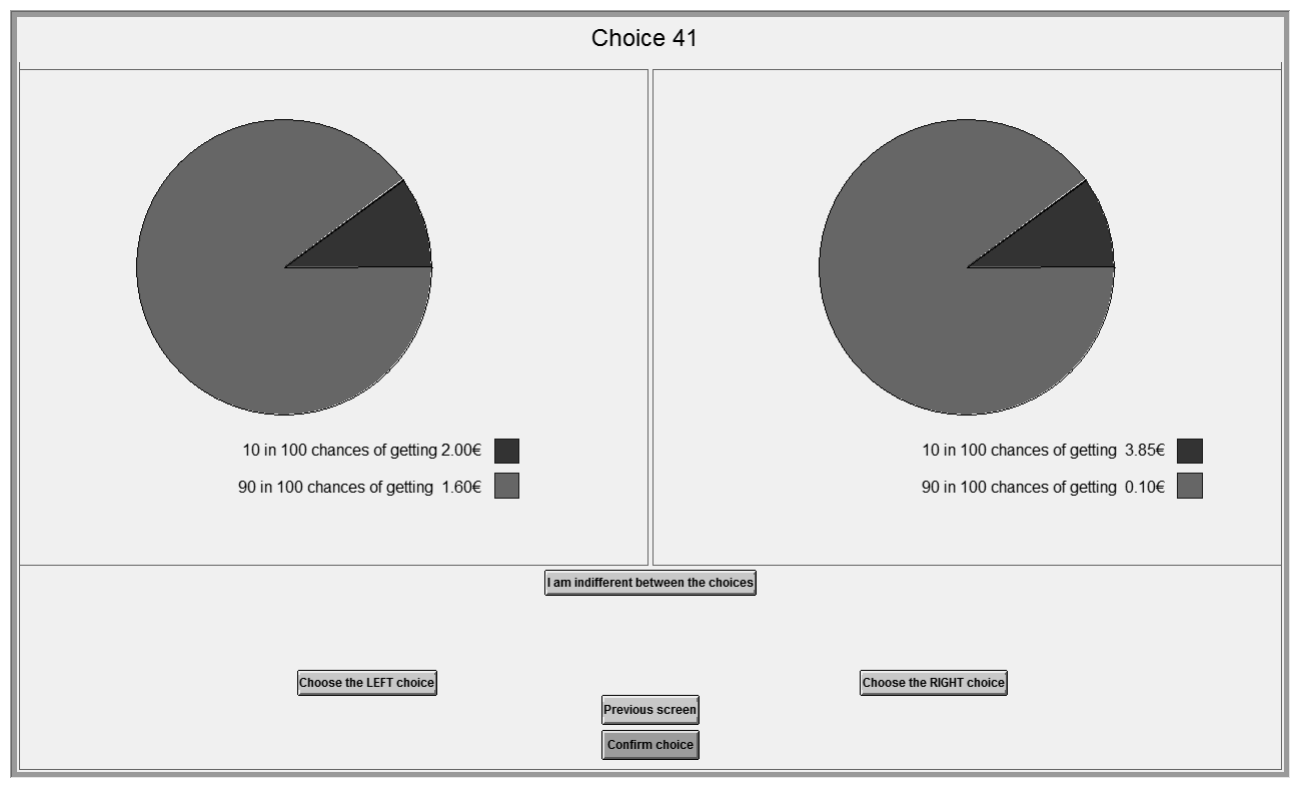
Example Decision Task.

Instead of providing a table of choices arrayed in an ordered manner all appearing at the same page as in H&L, each choice was presented separately showing probabilities and prizes [20]. Subjects could move back and forth between screens if they wanted to revise their choices. Once all ten choices in a table were made, the table was effectively inaccessible. In addition to the choices shown in Table 2, subjects also made a similar set of ten choices except the magnitudes of all payoffs were scaled up by a factor of five. The order of appearance of the set of ten choices (low versus high payouts) for each subject was completely randomized to avoid order effects [21]. An example of one of the decision tasks is shown in Figure 1. For each subject, one of the choices was randomly chosen and paid out.

### Conceptual specification: Expected utility versus Rank dependent utility theory

To estimate risk preferences, we follow the framework of [22]. Let the utility function be the constant relative risk aversion (CRRA) specification: 
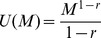
(1)where 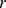 is the relative risk aversion (RRA) coefficient and where 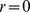 denotes risk neutral behavior, 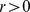 denotes risk aversion behavior and 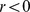 denotes risk loving behavior.

If we assume that EUT describes subjects risk preference tasks, then the expected utility of lottery *i* can be written as: 
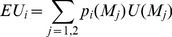
(2)where 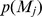 are the probabilities for each outcome 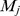 that are induced by the experimenter (i.e., columns 1, 3, 5 and 7 in Table 2).

Despite the intuitive and conceptual appeal of EUT, a number of experiments suggest that EUT often fails as a descriptive model of individual behavior. Although there are many proposed alternatives to EUT, here we consider RDU [23], which was incorporated into Tversky and Kahneman's [24] cumulative prospect theory. RDU extends the EUT model by allowing for non-linear probabilitiy weighting associated with lottery outcomes. To calculate decision weights under RDU, one replaces expected utility in equation (2) with: 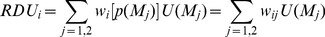
(3)where 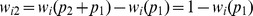 and 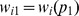 with outcomes ranked from worst (outcome 2) to best (outcome 1) and 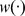 is the weighting function. We assume 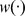 takes the form proposed by [24]:



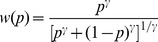
(4)When 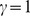, it implies that 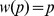 and this serves as a formal test of the hypothesis of no probability weighting.

### Stochastic error specifications

To explain choices between lotteries, one option is to utilize the stochastic specification originally suggested by [25] and popularized by [5]. In particular, the following index: 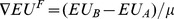
(5)can be calculated where 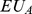 and 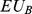 refer to expected utilities (or rank-dependent expected utilities) of options 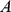 and 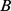 (the left and right lottery respectively, as presented to subjects), and where 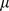 is a noise parameter that captures decision making errors. The latent index is linked to the observed choices using a cumulative standard normal distribution function 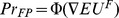, which transforms the argument into a probability statement. This is known as a Fechnerian (or Strong utility) model [26].

There are two observationally equivalent interpretations of the Fechner error specification. The most natural, given the set-up above, is that the term 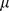 literally captures the effect of decision making errors on the part of the subjects. Another way to interpret this specification is through the random utility framework [27]. In this framework, utility consists of a systematic component, 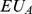, observable to the analyst, and a stochastic component, 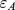, unobserved by the analyst but presumed known to the subject. In the random utility framework, the probability of choosing option A over B is the probability that 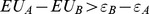. If the difference is distributed normally with mean zero and standard deviation 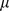, then the probability of choosing A over B is given by 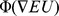 which, of course, is the same expression shown above.

An alternative to the error specification of equation (5) is: 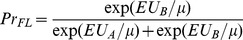
(6)


[13] notes that this form is similar to the form that have been used by [28] and [29]. It can easily be shown that this expression is algebraically identical to 
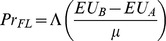
 [26] where 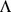 is the standard logistic distribution function with 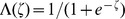. Therefore, although this is commonly referred to as the ‘Luce model’ in the respective literature [13], this is really the Fechnerian error (Strong utility) with a logit link. We will refer to these error specifications as 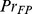 and 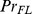 to denote the Fechnerian error with a probit and a logit link, respectively.

[3], [13] proposed a “contextual utility” error specification which modifies the error specifications in (5) and (6) to account for the range of possible outcome utilities. Contextual utility maintains that the error specification is mediated by the range of possible outcome utilities in a pair. The respective probability statements can be written as:
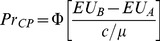
(7)and



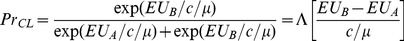
(8)In (7) and (8), 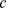 is a normalizing term, defined as the maximum utility over all prizes in a lottery pair minus the minimum utility over all prizes in the same lottery pair. It changes from lottery pair to lottery pair, and thus it is said to be contextual. Econometrically, the contextual utility correction is a way to accommodate lottery pair-specific heteroskedasticity. This type of heteroscedastic latent variable models are also called ‘moderate utility models’ [12], [13].

A third type of error models are due to [30] and have been popularized by [11]. In this case the probabilistic model can be written as:
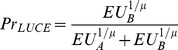
(9)which is algebraically identical to 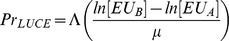. That is, the Luce model is like the Fechnerian (Strong utility) model but in which natural logarithms replace the utility values. This is also called a Strict Utility model in the respective literature [3], [12], [13]. Theorem 30 in [26] shows that “Any strict binary utility model is also a strong utility model, but not conversely”.

### Estimation

After defining the conceptual model and error specifications, the log-likelihood function can then be written as: 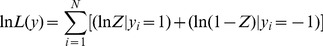
(10)where 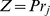 and 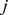 indexes the different error models 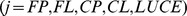. 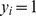 denotes the choice of the option B lottery and 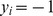 denotes the choice of the A lottery in the risk preference task 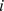. Subjects were allowed to express indifference between choices and were told that if that choice was selected to be played out, the computer would randomly choose one of the two options for them and that both choices had equal chances of being selected. The likelihood function for indifferent choices is constructed such that it implies a 50/50 mixture of the likelihood of choosing either lottery so that (10) can be rewritten as:



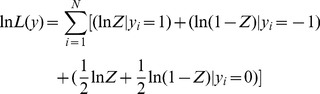
(11)


Equation (11) is maximized using standard numerical methods. The statistical specification also takes into account the multiple responses given by the same subject and allows for correlation between responses by clustering standard errors, which were computed using the delta method. Standard errors computed in Tables 3, 4 and 5, allow for intrasubject correlation to account for the fact that subjects made repeated choices and observations are not independent at the subject level. The robust estimator of variance that relaxes the assumption of independent observations involves a slight modiffication of the robust (or sandwich) estimator of variance ([31], pp. 295).

**Table 3:** Estimates assuming Fechner error with Probit and Logit link.

		Probit					Logit				
		Coef.	Std.Error	95% CI		Log-L	Coef.	Std.Error	95% CI		Log-L
EUT	r	0.682	0.050	0.584	0.780	−748.614	0.677	0.049	0.580	0.773	−738.096
	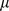	0.428	0.058	0.314	0.542		0.231	0.031	0.170	0.291	
	r	0.650	0.040	0.571	0.728	−747.947	0.638	0.040	0.561	0.716	−737.252
RDU	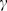	0.908	0.061	0.788	1.028		0.900	0.058	0.787	1.013	
	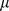	0.378	0.041	0.297	0.458		0.199	0.021	0.157	0.241	
	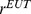	−0.633	0.269	−1.160	−0.105	−713.872	−0.832	0.331	−1.481	−0.183	−717.710
	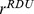	0.672	0.028	0.616	0.727		0.650	0.034	0.584	0.717	
Mixture	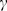	0.881	0.062	0.758	1.003		0.867	0.060	0.749	0.985	
	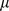	0.229	0.023	0.183	0.275		0.132	0.016	0.100	0.163	
	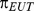	0.142	0.041	0.061	0.223		0.124	0.041	0.043	0.205	
	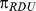	0.858	0.041	0.777	0.939		0.876	0.041	0.795	0.957	
Wald tests:											
RDU	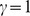	0.135					0.083				
Mixture	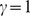	0.057					0.028				
Mixture	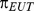 = 1 & 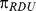 = 0	0.000					0.000				
Mixture	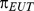 = 0 & 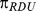 = 1	0.001					0.003				
Mixture	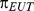 = 0 & 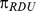 = 0	0.000					0.000				

Notes: EUT stands for expected utility theory, RDU stands for rank dependent utility. Bottom panel displays p-values from a Wald test for the respective hypothesis test.

**Table 4:** Estimates assuming Contextual utility with Probit and Logit link.

		Probit					Logit				
		Coef.	Std.Error	95% CI		Log-L	Coef.	Std.Error	95% CI		Log-L
EUT	r	0.580	0.060	0.462	0.697	−723.632	0.598	0.060	0.480	0.717	−719.187
	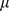	0.242	0.020	0.203	0.280		0.138	0.012	0.114	0.162	
	r	−0.038	0.105	−0.244	0.168	−702.763	−0.016	0.121	−0.254	0.222	−705.638
RDU	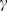	3.345	0.350	2.659	4.032		3.275	0.367	2.556	3.994	
	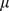	0.274	0.014	0.246	0.302		0.163	0.010	0.144	0.181	
	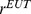	0.409	0.081	0.251	0.566	−693.940	0.084	0.192	−0.292	0.460	−696.607
	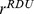	−0.291	0.173	−0.630	0.047		0.059	0.125	−0.186	0.304	
Mixture	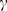	0.391	0.036	0.322	0.461		0.508	0.029	0.451	0.565	
	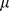	0.106	0.017	0.073	0.140		0.071	0.012	0.048	0.094	
	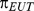	0.316	0.098	0.124	0.509		0.064	0.178	−0.285	0.412	
	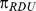	0.684	0.098	0.491	0.876		0.936	0.178	0.588	1.285	
Wald tests:											
RDU	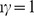	0.000					0.000				
Mixture	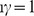	0.000					0.000				
Mixture	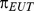 = 1 & 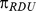 = 0	0.000					0.000				
Mixture	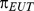 = 0 & 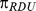 = 1	0.001					0.721				
Mixture	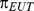 = 0 & 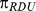 = 0	0.061					0.014				

Notes: EUT stands for expected utility theory, RDU stands for rank dependent utility. Bottom panel displays p-values from a Wald test for the respective hypothesis test.

**Table 5:** Estimates assuming Luce error (Strict utility).

		Coef.	Std.Error	95% CI		Log-L
EUT	r	0.603	0.079	0.447	0.759	−748.115
	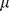	0.172	0.036	0.102	0.242	
	r	−0.500	0.179	−0.851	−0.148	−696.506
RDU	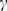	0.373	0.035	0.305	0.441	
	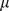	0.148	0.011	0.126	0.170	
	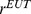	0.170	0.073	0.026	0.313	−695.669
	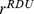	−0.806	0.241	−1.278	−0.334	
Mixture	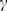	0.314	0.036	0.242	0.385	
	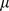	0.146	0.011	0.124	0.168	
	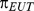	0.186	0.116	−0.041	0.414	
	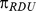	0.814	0.116	0.586	1.041	
Wald tests:						
RDU	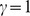	0.000				
Mixture	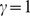	0.000				
Mixture	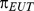 = 1 & 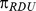 = 0	0.000				
Mixture	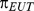 = 0 & 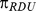 = 1	0.108				
Mixture	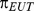 = 0 & 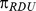 = 0	0.007				

Notes: EUT stands for expected utility theory, RDU stands for rank dependent utility. Bottom panel displays p-values from a Wald test for the respective hypothesis test.

Instead of discriminating between EUT and RDU models, one could allow the data generating process to admit more than one choice models. [14] and [15] allowed more than one process to explain observed behavior instead of assuming that the data are generated by a single process. [14] estimated a model where some choices were allowed to be EUT-consistent and other choices were allowed to be Prospect Theory-consistent (which is also equivalent to the rank dependent model in our experimental design) and found roughly equal support. [15] found that 20% of their subjects were behaving according to EUT while 80% were RDU maximizers. A mixture model poses a different question to the data. As [32] noted, “if two data-generating processes are allowed to account for the data, what fraction is attributable to each, and what are the estimated parameter values?” With the mixture specification we adopt, *choices* as opposed to *subjects* are categorized as completely EUT or RDU. Although it is possible to rewrite the likelihood in equation (11) such that the mixture is defined over subjects, [14] discuss how allowing choices across the same subject to differ, is consistent with experimental evidence that task domain can influence the strength of support for EUT. Similarly, our approach allows us being agnostic about the interpretation of the mixing probability. Note that the mixture specification in [15] only allows *subjects* to be completely categorized as EUT or RDU, whereas the mixture specification in [14] allows complete categorization at the level of *choices*. To put it otherwise, [15] allow for heterogeneity of preference functionals across individuals while [14] allow for heterogeneity of preference functionals across choices.

Let 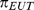 denote the probability that EUT is correct and 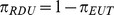 denote the probability that the RDU model is correct. We can then replace (11) with: 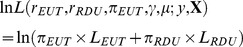
(12)


## Results

The purpose of this section is to demonstrate the implications of different assumptions about error specification and conceptual model, and illustrate how these choices can lead to significantly different characterizations of risk preferences; facts which make necessary the possibility to discriminate between models based on model fit criteria.

Tables 3, 4 and 5 show the estimated parameters from the EUT, RDU and mixture models when we assume Fechner error (with Probit and Logit link, respectively), contextual utility (with Probit and Logit link, respectively) or the Luce error (Strict utility). First compare the conceptual models, EUT and RDU, under the assumption of a Fehcner error specification without accounting for contextual utility (Table 3). Results show that subjects are on average risk averse (estimates of 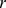 span between 0.638 to 0.682) and that the introduction of probability weighting does not have a significant effect on risk aversion. This is mainly because the estimate for 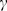 in the probability weighting function of the RDU model is very close to 1. In fact, Wald tests of 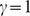 do not reject the null at the 5% level (shown at the lower panel of Table 3). Second, results between the probit and logit link in Table 3 are roughly the same. This is not surprising; it is pretty well known [33, for instance] that estimated parameters from a probit and a logit model differ up to a scale. As a practical note, one can use this result to check for convergence problems. If the Probit and Logit link produce strikingly different solutions, this is evidence that local maxima have been found. Note, that this equivalence between estimated parameters doesn't have to apply for mixture specifications since the estimated mixture probabilities in equation (12) are not included in the Probit or Logit link. Thus, in the context of EUT and RDU with a Fechner error, the choice between a Probit or a Logit link does not seem to have a substantive effect on implied risk preferences.

When we consider the mixture model for the Fechner error story, dramatically shifts in implied risk preferences occur. First note, that the probit and logit link produce similar results. With respect to the mixture probabilities 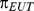 and 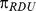, we find that roughly 14% and 12% of choices are explained by EUT (86% and 88% by RDU) in the probit and logit link, respectively. In addition, the estimated risk aversion coefficients imply risk loving preferences for EUT and risk aversion for RDU. Clearly, the results regarding underlying risk preferences are highly sensitivity to assumptions about heterogeneity of preference functionals.

**Figure 2:**
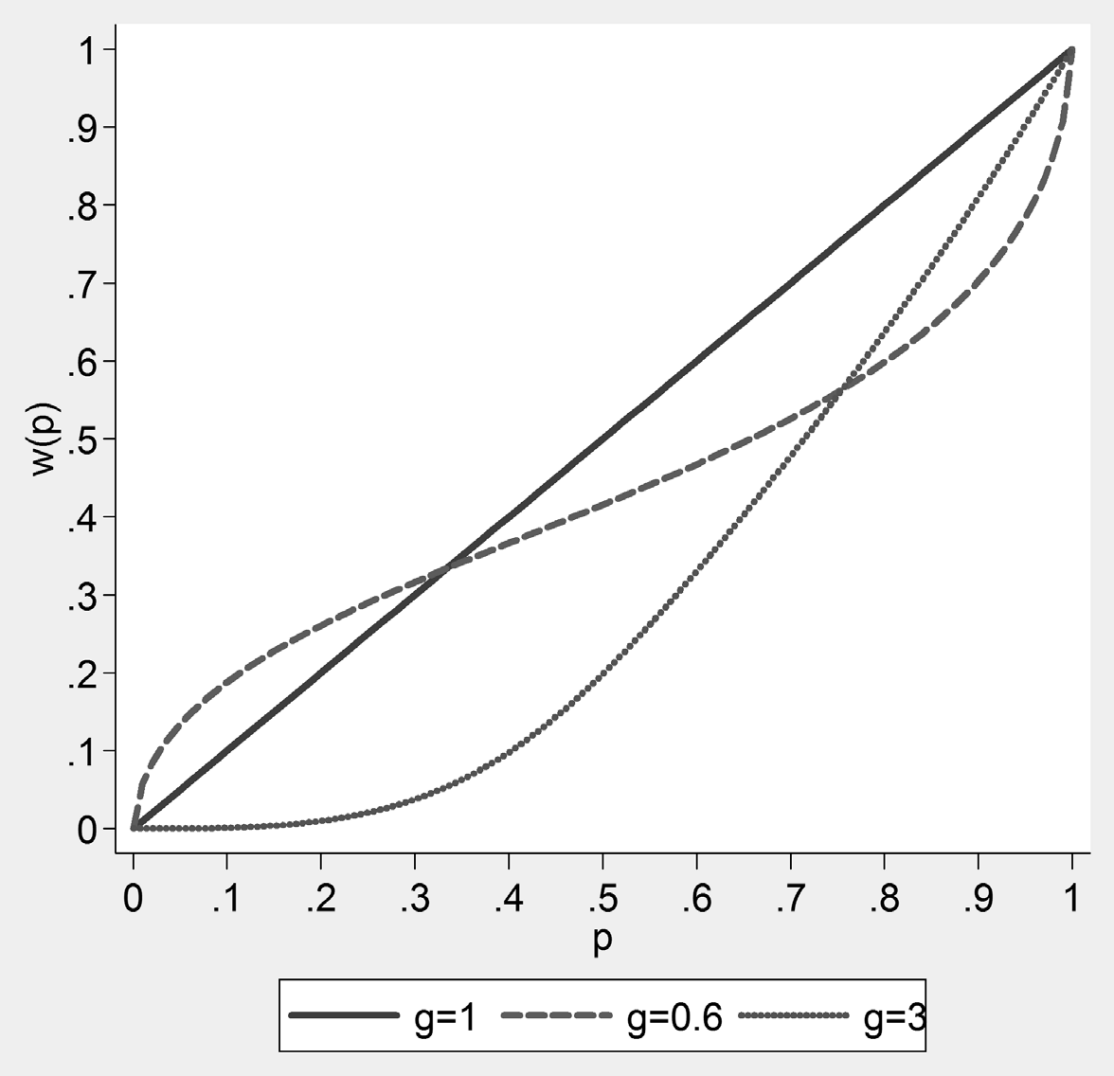
Comparison of probability weighting functions for three gamma (g) values.

Now we turn to the impact of contextual utility. The EUT model is least affected by the introduction of contextual utility in both the Probit and the Logit link. Although, the RRA coefficient estimates are lower in magnitude as compared to the non-contextual utility specifications (compare for example, the 0.58 estimate from Table 3 with 0.68 from Table 4 for the Probit link), the estimates still imply significant risk aversion. The most significant effects are found in the RDU specifications. RRA coefficients span around zero, implying risk neutrality, while 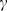 is estimated to have an unusually large value of 3. While large, this particular value for 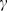, is not totally unrealistic, and Figure 2 shows it implies significant under-weighting for all probabilities. In fact, it implies that subjects totally ignore choices with probabilities lower than 0.2. The most commonly observed values for 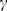, e.g. when 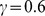, also imply under-weighting for probabilities larger than 0.35.

The introduction of a mixture specification not only produces different results as compared to the Fechner error counterparts, but it also produces different characterizations of risk preferences depending on whether the Probit or Logit links are assumed. For example, under contextual utility with a Probit link, the mixture probabilities imply that about 31.6% of all choices are EUT consistent while under the Logit link error only about 6% of the choices are consistent with EUT. In fact, the Wald test of whether 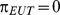 & 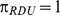 for the Logit link does not reject the null, implying that the mixture specification collapses to the non-mixture RDU specification. Under the contextual utility Probit link, the risk aversion coefficients imply risk aversion for EUT and risk neutrality for RDU while both RRA coefficient estimates under the contextual utility Logit link span around zero implying risk neutrality. Note that 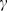 values are estimated at the more commonly observed values of 0.4 and 0.5 for the Probit and Logit links, respectively.

The last set of estimates using the Luce error produce even more striking differences in risk preference characterization. Table 5 shows that only risk preferences under EUT are consistent with results estimated with the Fechner error and contextual utility. The RDU specification implies significant risk loving behavior with 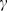 estimated at a value of 0.37 which highly contrasts the implied risk aversion from the RDU model of Table 3 (with a 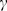 value close to 1) and risk neutrality of Table 4 with a 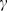 value of 3.3. When it comes to the mixture specification, mixture probabilities imply that the majority of choices are (81.4%) are RDU consistent. In fact, the Wald test of whether 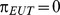 & 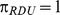 does not reject the null at the 5% level, implying that the mixture specification collapses to the non-mixture RDU specification. The estimates imply mild risk aversion for EUT choices and significant risk loving behavior for RDU choices.

Taken together, the results in Tables 3, 4 and 5 demonstrate that the menagerie of error stories that one could adopt for modeling risk preference estimation can lead to a variety of characterizations of risk preferences. Results from these tables show that implied risk preferences are consistent across error stories only under EUT, with estimated coefficients of risk aversion spanning the range of 0.58 to 0.68. However, under RDU the estimated coefficients of relative risk aversion span across models from a low of −0.50 (extreme risk seeking) to a high of 0.65 (extreme risk aversion). Moreover, the estimate of the shape of the probability weighting function under RDU goes from 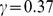 (extreme under-weighting of low probability events) to 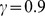 (near linear probability weighting implying EUT) to 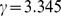 (under-weighting of all probabilities) depending on what is assumed about the error specification. The mixture specifications either imply that choices can be explained by both EUT and RDU or that in some cases RDU can better characterize all choices. The characterization of risk preferences varies as well. We find that the mixture of EUT-RDU can be characterized by several combinations like risk loving-risk aversion, risk aversion-risk neutrality, risk neutrality-risk neutrality or risk neutrality-risk loving preferences, depending on the error story. Thus, it is critically important to be able to select between competing models based on model fit criteria.

### Model selection criteria: Information criteria

Information criteria like the Akaikes Information Criterion (AIC) and the Bayesian Information Criterion (BIC) are common measures of goodness of fit; however, the statistics do not reveal how well a model fits the data in an absolute sense, i.e., there is no null hypothesis being tested. Nevertheless, these measures offer relative comparisons between models on the basis of information lost from using a model to represent the (unknown) true model.

Table 6 shows that based on AIC and BIC criteria, the contextual utility specifications are preferred over Fechner and Luce error stories within the EUT and mixture functionals, respectively. Within the RDU preference functional, the Luce error is preferred over contextual utility and Fechner error albeit contextual utility is the second best fitting model. When comparing between EUT, RDU and the mixture specifications, AIC indicates that the mixture model with contextual utility and a Probit link is the best fitting model whereas BIC indicates that RDU with Luce error is the best one. Note, that the second best model according to BIC coincides with the best model indicated by AIC (Mixture model with contextual utility and Probit link).

**Table 6:** Information criteria and out-of-sample Log-Likelihood function summary statistics.

			AIC	BIC	OSLLF
	Fechner error (Strong utility)	Probit	1501.227	1512.080	−759.043
		Logit	1480.192	1491.045	−747.636
EUT	Contextual utility	Probit	1451.263	1462.117	−733.911
		Logit	1442.374 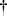	1453.228 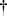	−729.002 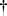
	Luce error (Strict utility)		1500.230	1511.083	−759.828
	Fechner error (Strong utility)	Probit	1501.894	1518.173	−759.351
		Logit	1480.504	1496.784	−747.694
RDU	Contextual utility	Probit	1411.525	1427.805	−714.008
		Logit	1417.276	1433.556	−715.762
	Luce error (Strict utility)		1399.013 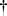	1415.293 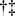	−707.149 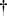
	Fechner error (Strong utility)	Probit	1437.744	1464.876	−724.826
		Logit	1445.420	1472.553	−731.082
Mixture	Contextual utility	Probit	1397.880 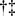	1425.013 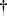	−705.556 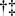
		Logit	1403.214	1430.346	−710.069
	Luce error (Strict utility)		1401.337	1428.470	−707.213

Notes: EUT stands for expected utility theory, RDU stands for rank dependent utility, AIC stands for Akaike information criterion, BIC stands for Bayesian information criterion, OSLLF stands for out-of-sample log-likelihood. 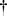 indicates best fitting error model within a preference functional. 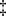 indicates best fitting error model across all preference functionals.

### Model selection criteria: Non-nested tests

The classical approach for testing between non-nested models is the Vuong test [34]. The Vuong test is a model selection test that compares between competing models and chooses the best model based on some predefined criteria. The Vuong test, as many other model selection criteria, is based on the Kullback-Leibler Information Criterion (KLIC), which measures the distance between a hypothesized likelihood function and the true likelihood function. The null hypothesis of the Vuong test is: 
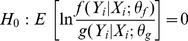
(13)where 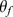 and 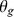 are parameters and 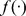, 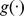 are the likelihood functions of the two competing models. The null in equation (13) implies that the two models are equivalent. The alternative hypothesis favors the model with the higher average log-likelihood, if it is significantly greater than the average log-likelihood of the competing model.

Because the Vuong test is only normally distributed asymptotically, distribution-free tests may be more desirable when the underlying population is not-normal. A non-parametric alternative to the Vuong test is the Clarke test [35]. The Clarke test is a paired sign test of the differences in the individual log-likelihoods from two non-nested models. The null hypothesis is that the probability of the log-likelihood paired differences being greater than zero is equal to the probability of the log-likelihood paired differences being less than zero, which in essence is a binomial test with 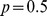. The Clarke test is similar to the Wilcoxon sign-rank test but without the additional assumption that the distribution of paired differences is symmetric.

If the models are equally close to the true specification, half the log-likelihood differences should be greater than zero and half should be less than zero. If one model is “better”, then more than half the log-likelihood differences should be greater than zero. The null hypothesis of the Clarke test is:
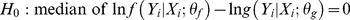
(14)


Top panel in Table 7 shows results from Vuong's tests which are performed between all possible pairs of error specifications for the EUT, RDU and the mixture models. A large positive value of the Vuong statistic (with a corresponding low p-value) favors the error model listed in the second column versus the model indicated in the top row. A large negative value (with a corresponding high p-value) favors the model listed in the top row versus the model indicated in the second column.

**Table 7:** Vuong's (top panel) and Clarke's non-parametric (bottom panel) non-nested tests.

Vuong's test									
		FP		FL		CP		CL	
		Vuong stat.	p-value	Vuong stat.	p-value	Vuong stat.	p-value	Vuong stat.	p-value
EUT	FL	5.024	0.000	-	-	-	-	-	-
	CP	3.324	0.000	2.137	0.016	-	-	-	-
	CL	**3.907**	**0.000**	**2.927**	**0.002**	**3.038**	**0.001**	-	-
	LUCE	0.068	0.473	−1.407	0.920	−6.588	1.000	**−6.427**	**1.000**
RDU	FL	4.998	0.000	-	-	-	-	-	-
	CP	4.251	0.000	3.827	0.000	-	-	-	-
	CL	3.922	0.000	3.451	0.000	-6.568	1.000	-	-
	LUCE	**5.548**	**0.000**	**5.083**	**0.000**	**1.818**	**0.035**	**2.456**	**0.007**
Mixture	FL	−1.681	0.954	-	-	-	-	-	-
	CP	**3.872**	**0.000**	**5.104**	**0.000**	-	-	-	-
	CL	3.152	0.001	3.838	0.000	−1.158	0.877	-	-
	LUCE	3.492	0.000	4.335	0.000	−0.979	0.836	0.623	0.267

Notes: EUT stands for expected utility theory, RDU stands for rank dependent utility. FP = Fechner error with Probit link, FL = Fechner error with Logit link, CP = Contextual utility with Probit link, CL = Contextual utility with Logit link, LUCE = Luce error (Strict utility)

The values shown in the bottom panel concerning Clarke's test are p-values for the hypothesis 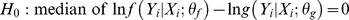 where 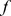 is the model labeled in the respective column and 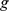 is the model labeled in the respective row. The ‘
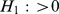
’ or the ‘
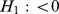
’ labels in the second row indicate which one-sided alternative are we testing for.

Within the EUT preference functional, the CL (contextual utility - Logit link) model is favored against all other models (as indicated by the corresponding Vuong statistics marked in bold). This is in agreement with the AIC/BIC measures in Table 6. Within the RDU functional, the Luce error is favored against all other models and is in agreement with the AIC/BIC measures. When it comes to the mixture specification, the CP (contextual utility - Probit link) model is favored when compared with the Fechner errors (in accordance with AIC/BIC measures) but does equally well when compared to the Luce error and contextual utility with Logit link (CL), albeit the corresponding p-values are not that far away from the 10% significance level (1−0.836 = 0.164 and 1−0.877 = 0.123, respectively) which would render the CP preferable. In all, results from Vuong's tests support the results from the AIC and BIC model selection criteria.

Vuong's test is suitable for non-nested models, thus we do not compare error specifications between EUT, RDU and the mixture models since these are, by construction, nested in each other. For example, one can test whether the mixture model collapses to EUT or RDU by testing whether the mixture probabilities are statistically significantly different from zero. Or one can test whether RDU collapses in EUT by testing whether 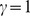. For the mixture CP model, Wald tests in Table 4 show that it neither collapses to either EUT or RDU, nor does RDU collapses to EUT.

Bottom panel in Table 7 shows results from Clarke's non-parametric test. For each preference functional (EUT, RDU, mixture), each error story is compared against all other error stories. Each comparison involves two, one-sided tests. Obviously, rejection of the null for one of the one-sided alternatives implies that we fail to reject the null for the other one-sided alternative. There will be cases where we fail to reject the null in both of the one-sided tests. This would imply that the error models that are being tested do equally well.

For the EUT preference functional the error model that stands out is the CL model. It is favored in three out of four comparisons (i.e., against FP, CP, LUCE) and does equally well with FL. However, FL only does well when in direct comparison with FP. A problem with this type of tests now becomes apparent. Pairwise comparisons do not necessarily obey a transitivity principle. More specifically, the problem here is that FL does equally well with CP, FL does equally well with CL while CL is preferred to CP. If there was transitivity, CL should do equally well to CP. Even so, the general notion is that the contextual utility with a Logit link is preferable, which is in accordance with all previous methods of model fit we have reviewed so far.

The RDU preference functional is even more interesting because the unanimous winner is the CP model (it is favored in four out of four comparisons) which contrasts results from fit criteria we reviewed so far. Note that AIC/BIC measures and the Vuong test were all in favor of the Luce model. This indicates that comparisons that are based solely on one model fit criterion are not guaranteed to be flawless.

For the mixture preference functional, the contextual utility with a Probit link is favored in four out of four comparisons, in accordance with results from previously discussed fit criteria.

### Model selection criteria: Out-of-sample predictions

The Out-Of-Sample Log Likelihood (OSLLF) criterion evaluates models by their fit out of sample. In essence, the OSLLF approach uses one set of data to estimate the parameters of the model, and then, given these parameters, calculates the likelihood function values observed at out-of-sample observations. The OSLLF value is calculated by using out-of sample observations to calculate the likelihood function: 
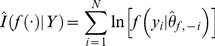
(15)where 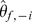 is the parameter vector estimated without the *i*th set of observations. The OSLLF value can be calculated in several ways [10]. The estimate 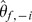 could be calculated using cross-validation where 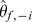 is estimated using every observation except *i*. This is referred to as “leave one out at a time forecasting.” Alternatively, one could partition the observations into groups where each group is iteratively omitted and 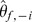 is estimated. Then, the omitted group of observations can be used to calculate the OSLLF. This procedure is known as grouped-cross-validation. In what follows, we carry out group-cross validation with individuals being the partitions, where each partition contains twenty observations (as many as the choices of the subject). Essentially, we leave one subject (and their associated 20 choices) out at a time, estimate the model, and calculate equation (15) for the subject. The process is repeated for every subject in the sample.

Table 6 reports OSLLF values for each of the error specification for each preference functional (EUT, RDU and the mixture model). When comparing within preference functionals the OSLLF agrees with both AIC and BIC measures. When comparing across all preference functionals, the mixture specification with contextual utility and a Probit link ranks highest. This is in accordance with AIC but not BIC, albeit, as noted before, the mixture CP model is the second best model in terms of BIC.

Combining the insights gained from the different fit criteria and tests reviewed above, we can more safely conclude that a mixture specification with contextual utility and a Probit link fits the data better than any other preference functional and error story examined in this paper. This is reinforced by the fact that the mixture CP model does not collapse either to EUT or RDU, nor RDU collapses to EUT, as indicated by the corresponding Wald tests in Table 4.

## Discussion

To derive estimates of individuals risk preferences, analysts have to have some mechanism for translating the conceptual models of risky decision making into an empirical model that includes stochastic errors. The results presented in this paper reveal that seemingly innocuous assumptions about this stochastic process can lead to substantially different inferences about risk preferences. Indeed, one can estimate parameters consistent with a high level of risk seeking or a high level of risk aversion depending on how errors are incorporated into the statistical model; a finding which suggests caution in naively assuming adopting a single error specification.

A battery of in- and out-of-sample model selection criteria suggest that the model that best fits our data is an EUT-RDU mixture model assuming contextual utility with a Probit link. We find that 31.6% of the sample is characterized by EUT with a coefficient of relative risk aversion equal to 0.41, and 68.4% is characterized by RDU with a coefficient of relative risk aversion statistically indistinguishable from zero but with a probability weighting function implying significant overweighting of low probability outcomes and under-weighting of moderate to high probability outcomes.

In our attempt to provide a battery of model fit selection criteria, we have only focused on the specific preference functionals and error specifications that are likely to be considered the main contenders. This was not an exhaustive test of the full menagerie of preference functionals or stochastic errors that exist in the literature nor could such an exercise be covered in a single paper. Researchers would need to apply the methods discussed herein in their problem at hand.
